# The Role of Prophylactic and Adjuvant Hyperthermic Intraperitoneal Chemotherapy (HIPEC) in Prevention of Peritoneal Metastases in Advanced Colorectal Cancer

**DOI:** 10.3390/jcm12206443

**Published:** 2023-10-10

**Authors:** Beatrice J. Sun, Sara K. Daniel, Byrne Lee

**Affiliations:** Section of Surgical Oncology, Department of Surgery, Stanford University School of Medicine, Stanford, CA 94305, USA; sunbj@stanford.edu (B.J.S.); skdaniel@stanford.edu (S.K.D.)

**Keywords:** prophylactic HIPEC, adjuvant HIPEC, advanced colorectal cancer, peritoneal metastases, locoregional recurrence

## Abstract

Hyperthermic intraperitoneal chemotherapy (HIPEC) is a locoregional therapy that may be combined with cytoreductive surgery (CRS) to treat patients with colorectal cancer and peritoneal metastases (PM). In recent years, three randomized controlled trials (RCTs) have investigated the role of prophylactic or adjuvant HIPEC in preventing the development of PM in patients with high-risk colorectal cancer: PROPHYLOCHIP and COLOPEC evaluated adjuvant HIPEC, and HIPECT4 studied concurrent HIPEC and CRS. Although PROPHYLOCHIP and COLOPEC were negative trials, a great deal may be learned from their methodology, outcome measures, and patient selection criteria. HIPECT4 is the first RCT to show a clinical benefit of HIPEC in high-risk T4 colorectal cancer, demonstrating improved locoregional disease control with the addition of HIPEC to CRS with no increase in the rate of complications. This review critically examines the strengths and limitations of each major trial and discusses their potential impact on the practice of HIPEC. Several additional ongoing clinical trials also seek to investigate the role of HIPEC in preventing PM in advanced colorectal cancer.

## 1. Introduction

Colorectal cancer is the third most common neoplasm worldwide with an estimated 1.9 million new cases in 2020 [1]. Peritoneal metastases (PM) are reported in up to 10% of all patients, although the incidence is likely underestimated due to the limited sensitivity of current diagnostic imaging in detecting peritoneal disease [2,3]. Patients with colorectal cancer metastatic to the peritoneum demonstrate a poor response to systemic chemotherapy and have significantly lower overall survival compared to those with isolated metastases to non-peritoneal sites [4,5]. Thus, locoregional therapies, including cytoreductive surgery (CRS) and hyperthermic intraperitoneal chemotherapy (HIPEC), have emerged in recent decades, and despite a negative clinical trial result from PRODIGE 7 [6], CRS–HIPEC has remained a promising therapeutic option for colorectal cancer with PM [7,8,9,10].

A number of risk factors have been associated with the development of PM in colorectal cancer, including previously resected synchronous PM, ovarian metastases, perforated primary tumor, locally advanced tumor (T4), and mucinous or signet ring cell histology [11,12]. In these high-risk patient populations, proactive HIPEC administered either during primary oncologic resection or during a second-look operation is being investigated as a strategy to prevent or treat early peritoneal disease. Several cohort studies have shown encouraging results thus far. Sammartino et al. compared CRS–HIPEC with standard colorectal resection for patients with locally advanced disease and showed that CRS–HIPEC was associated with a significantly lower rate of PM (4% vs. 22%, *p* < 0.01) [13]. Long-term follow up also suggested improved disease-free and overall survival in those who underwent HIPEC [14]. Another study revealed consistent findings that favor the use of HIPEC in high-risk patients with colorectal cancer with 9% vs. 43% (*p* < 0.004) developing PM at 5 years [15]. Additionally, second-look surgery, performed weeks to months after the initial surgical resection, has been proposed to help detect and treat subclinical PM early. When combined with adjuvant HIPEC, this strategy has the potential to decrease PM and improve survival outcomes [16,17].

Despite the encouraging results from these cohort studies, questions remain regarding the efficacy of HIPEC in preventing PM for high-risk colorectal cancer. Three important randomized controlled trials (RCTs) have assessed the use of adjuvant and prophylactic HIPEC in this patient population (Table 1). We will review the results of these trials and discuss their overall impact on the future landscape of CRS and HIPEC in patients with colorectal cancer at high risk for PM.

## 2. PROPHYLOCHIP-PRODIGE 15 (France, 2020)

### 2.1. Trial Design and Results

**Figure 1:**
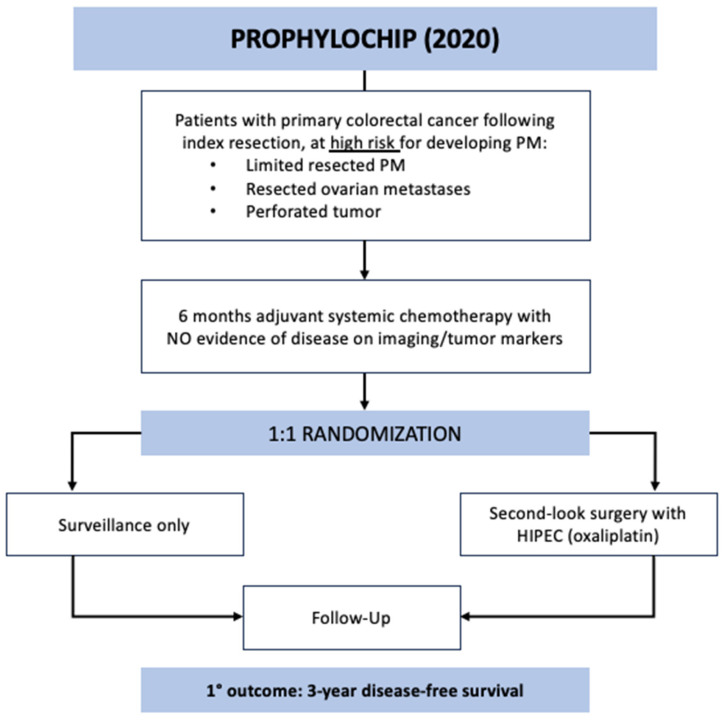
PROPHYLOCHIP trial schematic.

The PROPHYLOCHIP trial was a multicenter, phase 3 study that enrolled patients across 23 hospitals in France from June 2010 to March 2015 [18]. The study aim was to evaluate the potential survival benefit of systematic second-look surgery and HIPEC in patients with colorectal cancer at high risk of developing PM, which was defined as localized peritoneal disease resected at index operation, resected ovarian metastases, or perforated tumor. All patients underwent surgical resection of their primary tumor followed by 6 months of adjuvant systemic chemotherapy. If there was no evidence of recurrence at 6 months on imaging or tumor markers (*n* = 150), they were randomized 1:1 to the surveillance or treatment group (Figure 1). The treatment arm underwent second-look surgery that consisted of exploratory laparotomy followed by HIPEC administration with oxaliplatin for 30 min (IP oxaliplatin 460 mg/m^2^ alone or oxaliplatin 300–360 mg/m^2^ plus irinotecan 200 mg/m^2^ with concurrent IV 5-FU 400 mg/m^2^ and leucovorin 20 mg/m^2^). Over half of these patients were found to have macroscopic PM during second-look surgery and underwent CRS prior to HIPEC. All study patients were followed every 3 months with a physical exam, CT imaging, and tumor markers. The primary outcome of PROPHYLOCHIP was 3-year disease-free survival (DFS) or time from randomization to PM, distant recurrence, or death by any cause. There was no difference in the 3-year DFS between the treatment and surveillance groups (44% vs. 53%; HR 0.97, CI 0.61–1.56, *p* = 0.82). Moreover, no difference was detected in secondary outcomes, including 3-year peritoneal recurrence-free survival (59% treatment vs. 61% surveillance) and 5-year overall survival (OS) (68% treatment vs. 72% surveillance). Notably, the major postoperative complication rate (grade 3–4) after second-look surgery was high at 41% (29/71 patients), including 17% (12/71) with intra-abdominal complications, 67% (8/12) of whom required reoperation. Additionally, 43 patients in total developed recurrence amenable to surgery during the follow-up period, 35 of whom underwent repeat CRS with or without HIPEC.

### 2.2. Strengths and Contributions

The PROPHYLOCHIP population encompassed several of the previously described high-risk factors for developing future PM, including localized and resected peritoneal disease, resected ovarian metastases, and perforated tumors [11]. Despite complete cytoreduction, adjuvant systemic chemotherapy, and no evidence of disease with imaging or tumor markers, over 50% of these patients were found to have macroscopic PM at the second-look operation. This study demonstrates the propensity for high-risk patients, particularly those with resected peritoneal disease and ovarian metastases, to develop occult short-interval peritoneal recurrence and highlights the limitations of conventional surveillance methods. It also highlights the importance of studying proactive treatment strategies to prevent peritoneal recurrence in these patients.

### 2.3. Limitations and Discussion

Although PROPHYLOCHIP was one of the first randomized controlled trials to investigate HIPEC in the setting of high-risk colorectal cancer, the study has several limitations.

Oxaliplatin as a HIPEC agent has been commonly used in Europe [19] due to its efficacy in patients with colorectal cancer and PM, demonstrated in retrospective studies [20,21,22]. However, PRODIGE 7, the only RCT to evaluate HIPEC in the treatment of colorectal cancer with PM, did not show a survival benefit of CRS with oxaliplatin-based HIPEC compared to CRS alone [6]. PROPHYLOCHIP utilized the same oxaliplatin-based regimen and schema, which may not be an effective perfusion strategy. One reason for this regimen’s failure is the potential chemoresistance to intraperitoneal oxaliplatin induced from systemic oxaliplatin therapy. A study that evaluated the chemosensitivity of tumor tissue before and after HIPEC with oxaliplatin showed a 61% resistance to HIPEC in those who had received a neoadjuvant oxaliplatin-containing regimen compared to 33% resistance in those who did not [23]. Furthermore, an ex-vivo study by Nagourney et al. demonstrated a significantly higher concentration of oxaliplatin was required to achieve tumor cell death in those who were pretreated with an oxaliplatin-based therapy, particularly if treated within 2 months [24]. In this context, 89% of the patients in PROPHYLOCHIP who underwent second-look surgery with HIPEC had received prior oxaliplatin-based chemotherapy (FOLFOX or XELOX), which may have reduced the efficacy of HIPEC. Another criticism is the short perfusion time of 30 min (rather than the typical 90 min), which may limit both the absorption and cytotoxic effect of oxaliplatin on cancer cells [19,25].

The concept of second-look surgery as described in the study is not new and has been described in prior studies leading up to this trial. An advantage of a second-look operation 6 to 12 months after the initial resection in high-risk patients is the ability to diagnose subclinical PM not detected on imaging in order to surgically treat limited PM early; the addition of HIPEC during second look may further decrease the recurrence rates of PM [17,26,27,28]. Although the efficacy of HIPEC at the time of initial CRS has not been directly compared to HIPEC after second-look surgery, the latter may carry increased risks. In PROPHYLOCHIP, the patients in the second-look group with high-risk features received no IP chemotherapy after the initial surgery but did undergo 6 months of adjuvant chemotherapy [18]. There is typically a delay of 6 to 12 weeks between surgery and chemotherapy initiation [29,30], during which time microscopic disease within the peritoneal cavity may continue to grow and exhibit limited sensitivity to systemic therapy [5,31]. In this trial, second-look surgery was performed via open laparotomy for complete exploration, including the reopening of previous dissection planes. The resultant complication rate was quite high at 41% [18]. Not only is this significantly higher than the complication rates reported in the literature for CRS–HIPEC [32,33,34], but the second-look operation in this study was performed with preventive intent in asymptomatic patients with no preoperative evidence of disease. High morbidity and extended recovery may introduce more harm than benefit to these patients and, thus, should not be accepted. Furthermore, 30% of second-look operations with macroscopic PM were found to be non-malignant on pathology, suggesting the possibility of overtreatment of subclinical lesions during a second-look exploration [35].

The study included patients with localized peritoneal disease and ovarian metastases that were resected at the initial surgery as well as those with perforated tumors in the absence of peritoneal disease. Although all are considered high-risk, these patients represent a heterogeneous group: those with resected peritoneal and ovarian metastases have distant metastatic (M1) disease, whereas patients with perforated tumors (without PM) have locally advanced, non-metastatic disease (T4M0) [36]. As described in the literature, the estimated peritoneal recurrence on surveillance is 54–75% after resection of limited synchronous PM, 56–62% after resection of ovarian metastases, and 14–54% after primary tumor perforation [11,27]. The comparatively lower rate of PM after tumor perforation is expected due to the underlying pathophysiology of peritoneal dissemination. On a molecular level, the presence of macroscopic peritoneal or ovarian metastasis indicates that tumor cells have already left the primary site and disseminated across the peritoneal cavity to seed and invade the peritoneum or ovary [37]. In this context, even when visible M1 disease is completely resected, there is often additional microscopic peritoneal disease that will grow if left inadequately treated. In perforated tumors, however, cancer cells have separated from the primary tumor but may not have seeded the peritoneum and should have a lower risk of developing into PM [37]. Thus, the rationale for HIPEC in the setting of resected M1 disease is to treat disease that has already seeded into the peritoneum and may be regarded as therapeutic, whereas the rationale for HIPEC in the context of a perforated tumor without PM is to treat free cancer cells in the peritoneal cavity to *prevent* them from seeding the peritoneum and may be regarded as prophylactic against the development of PM. As such, the patient population with resected metastases is inherently different from those with perforated tumors and should be studied as separate cohorts in the future.

In this trial, the primary outcome was defined as 3-year DFS, which combines the time to peritoneal recurrence, distant metastases, and death from any cause. The rationale for HIPEC is for use as a locoregional therapy to target disease in the peritoneum; due to the presence of the peritoneal–plasma barrier, intraperitoneal chemotherapy reaches higher concentrations within the peritoneum with reduced systemic absorption and toxicity compared to systemic chemotherapy. The addition of hyperthermia acts synergistically to mediate cytotoxicity to the intraperitoneal cancer cells [38,39,40]. While the mechanism of HIPEC has been shown to decrease local peritoneal recurrence [13,15,41], it does not impact distant metastases or overall survival [13,42]. This is likely because metastases to non-peritoneal sites, such as the liver and lung, occur hematogenously via the portal and systemic circulation, respectively, and the minimal systemic uptake of chemotherapy during HIPEC does not significantly affect this route of disease spread [42,43]. Therefore, outcomes evaluating the efficacy of HIPEC should be focused on local peritoneal recurrence rather than measures related to non-peritoneal recurrence.

In PROPHYLOCHIP, after 6 months of adjuvant chemotherapy with no evidence of disease recurrence, the experimental group exhibited a 52% rate of macroscopic PM at second look, 70% of which were confirmed on pathology to be malignant. While the surveillance group did not undergo invasive procedures to assess PM, it is reasonable to hypothesize that a similar rate of occult PM would be present. This again highlights the poor sensitivity of cross-sectional imaging in detecting peritoneal disease, particularly lesions smaller than 5 mm, yet CT imaging with intravenous contrast remains the overall imaging modality of choice and was used for surveillance in this trial [3,44,45]. Additionally, 47% of all patients experienced recurrent disease during the follow-up period, and 23% underwent further resection with or without HIPEC. This indicates that the patients in this trial were followed closely and treated at early stages for their recurrent disease, but the long-term outcomes cannot be attributed to only the primary trial intervention. Even though the patients undergoing repeat resection are censored from the Kaplan–Meier curve in Figure 3 of the PROPHYLOCHIP trial [18], overall survival should still be interpreted with caution as a large proportion of patients were censored in the analysis.

## 3. COLOPEC (The Netherlands, 2019)

### 3.1. Trial Design and Results

**Figure 2:**
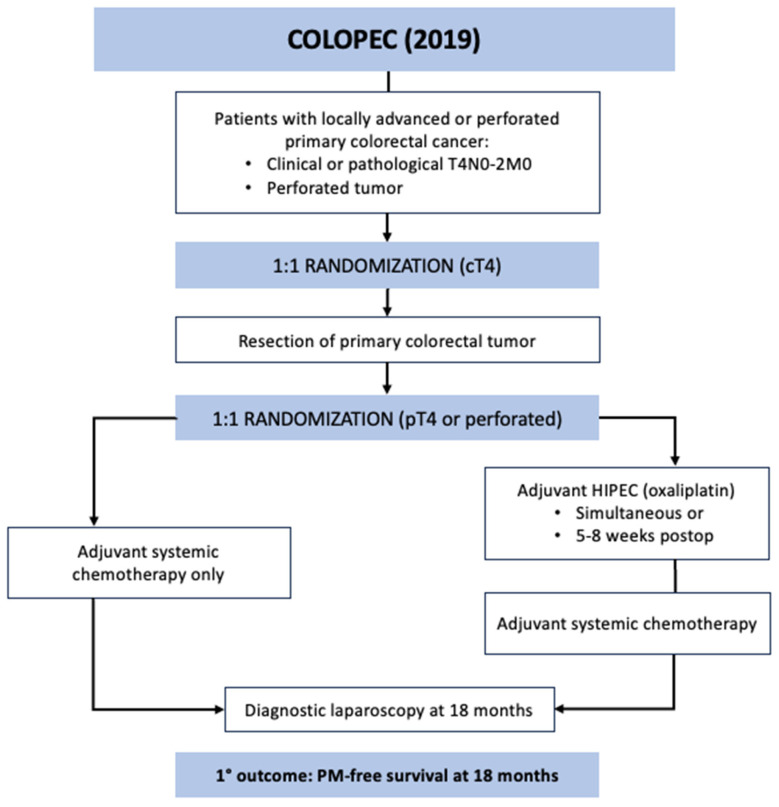
COLOPEC trial schematic.

COLOPEC was a phase 3 RCT conducted across nine Dutch hospitals specializing in HIPEC between April 2015 and February 2017 [30]. The study aimed to evaluate the efficacy of adjuvant HIPEC with oxaliplatin after curative-intent resection in patients with locally advanced colorectal cancer. The included patients had T4N0-2M0 or perforated tumors without evidence of PM. A minority of the patients were randomized prior to primary tumor resection based on clinical T4 tumor (*n* = 30), and the majority were randomized after surgical resection following pathological confirmation of T4 or perforated tumor (*n* = 172) (Figure 2). The experimental arm underwent simultaneous or adjuvant HIPEC at 5–8 weeks after the index operation with the following regimen: IP oxaliplatin 460 mg/m^2^ for 30 min with concurrent IV 5-FU 400 mg/m^2^ and leucovorin 20 mg/m^2^ followed by 6 months of systemic chemotherapy. The control arm underwent systemic chemotherapy only. Overall, 87% of the study patients started chemotherapy at a median time of 6 weeks and 10 weeks after primary resection in the control and experimental groups, respectively. The primary endpoint was PM-free survival at 18 months, which was assessed with diagnostic laparoscopy in the patients with no imaging evidence of recurrent disease. No difference in PM-free survival was detected on intention-to-treat analysis between the groups at 18 months: 80.9% in the experimental vs. 76.2% in the control groups (one-sided log rank *p* = 0.28). Morbidity in the adjuvant HIPEC cohort was 14%, and the 18-month overall survival was similar between the groups at 93%. During the study period, 19 patients developed PM in the experimental arm: 9 at surgical exploration prior to HIPEC, 8 during routine follow up, and 2 during laparoscopy at 18 months. In the control arm, 23 patients developed PM: 16 during routine follow up and 7 during diagnostic laparoscopy. In the patients who developed PM, 13 of 19 (68%) in the experimental group and 15 of 23 (65%) in the control group were treated with CRS and HIPEC using mitomycin.

### 3.2. Strengths and Contributions

The COLOPEC trial represented a national effort across several institutions in the Netherlands. Compared to PROPHYLOCHIP, the patient population was more homogenous as only patients with locally advanced disease were included, and those found to have PM at the initial surgery or time of adjuvant HIPEC were excluded. One advantage of adjuvant HIPEC in this trial was that it allowed for the inclusion of perforated tumors that required an emergent operation for initial surgical resection; these would have been excluded from the study if HIPEC had been concurrent.

A strength of the study is its use of PM-free survival as the primary outcome, which is a good surrogate for HIPEC as a locoregional therapy rather than a systemic treatment. Given the limited sensitivity of imaging in the detection of PM, the study utilized diagnostic laparoscopy to determine the presence of PM at the 18-month follow-up period.

### 3.3. Limitations and Discussion

As in PROPHYLOCHIP, the COLOPEC trial used an oxaliplatin-based HIPEC protocol, which is limited by a short perfusion duration, oxaliplatin-resistance from preoperative systemic chemotherapy, and poor efficacy thus far in trials for treatment of PM from colorectal cancer, as previously discussed [6,18,19,24].

The timing of adjuvant HIPEC in COLOPEC introduces additional limitations. In contrast to PROPHYLOCHIP in which the study arm underwent 6 months of systemic chemotherapy after the initial resection prior to consideration for second-look laparotomy with HIPEC, those in COLOPEC largely underwent staged HIPEC at 5–8 weeks after the initial surgery. A major challenge to undergoing repeat surgical intervention within such a short time interval, particularly when approximately half the initial operations were performed open, is the burden of adhesions [46]. The pathophysiology of postoperative adhesions is related to the inflammatory response to surgery, resulting in excess fibrin deposition that serves as a scaffold for fibroblasts to produce fibrous scar tissue at 4–8 weeks [47,48]; additional risk factors for adhesion formation include lower gastrointestinal procedures and open surgery [46,47,49,50]. In the COLOPEC trial, the rate of adhesions at the time of adjuvant HIPEC was significant: 66% of patients were found to have adhesions, including 34% with moderate to extensive adhesive burden. Adhesions not only increase operative time, technical difficulty, and risk of complications that include missed enterotomy, but the presence of adhesions also prevents full exposure of previous dissection planes, which may harbor residual or microscopic cancer cells in the peritoneal cavity, thereby limiting the efficacy of HIPEC [39,51,52,53,54]. Although most patients underwent laparoscopic HIPEC, 8% required conversion to open, and in total, 34% underwent open laparotomy for HIPEC perfusion only. This represents a highly invasive second operation and hospital admission within 2 months, especially in the absence of concurrent surgical resection. Furthermore, a second operation resulted in a 4-week delay in initiating systemic chemotherapy. All these technical and patient factors should be considered and suggest several advantages of administering simultaneous HIPEC in the prophylactic rather than adjuvant setting. Interestingly, a minority of patients in the study did undergo simultaneous HIPEC (9%); however, due to the small number, this was inadequate for a sub-analysis.

The randomization in COLOPEC was not unified: 30 patients with clinical T4 tumors were randomized preoperatively, whereas 172 were randomized postoperatively after confirmation of T4 tumor or perforation on pathology. As a result of this, the trial’s intention-to-treat analysis included those who were clinically lower stage (cT1–T3, over 30% in each arm) as well as patients who were pathologically lower stage (pT2–T3, 13% in each group), which may limit the generalizability of the findings to the intended patient population with T4 and perforated colorectal tumors. This also suggests overtreatment of those with pT2 or pT3 tumors as they do not carry the same risk of developing PC as those with pT4 or perforated tumors (8–19% and 14–54%, respectively) [11,27] yet were subject to a second operation for HIPEC, additional hospitalization, and potential for complications [30].

It is also worth noting that approximately 10% of the patients in the experimental arm exhibited PM within 2 months at the time of their second operation for adjuvant HIPEC. Such early interval peritoneal spread suggests the possibility that PM were not identified at the initial operation or there was early disease progression due to tumor manipulation or spillage during surgery [55,56]. This calls into consideration the potential benefit of a more radical initial operation (which may include omentectomy, lymphadenectomy, and/or oophorectomy) and upfront HIPEC [41]. Although these trial patients who were found to have early PM did not receive adjuvant HIPEC according to the protocol, they did undergo CRS–HIPEC for treatment of their peritoneal disease. In fact, during the study period, 13 patients with PM in the experimental group and 15 in the control group were treated with off-protocol CRS–HIPEC. In contrast to the study regimen, these patients received HIPEC with mitomycin C regardless of whether they had been previously perfused with oxaliplatin as part of the trial. The authors performed an intention-to-treat analysis, which included 13% of the trial arm who never received the experimental treatment (adjuvant HIPEC) and 14% of the total study group who underwent CRS–HIPEC for PM off-protocol [57]. This demonstrates that a significant proportion of the patients included in the analysis were not the intended population for the trial, and thus, the results should be interpreted with caution.

Finally, the primary outcome of PM-free disease was measured with diagnostic laparoscopy at 18 months. While the advantages of diagnostic laparoscopy are direct visualization of the peritoneal cavity and improved sensitivity of detecting PM compared to imaging [58,59], the drawbacks of the procedure are that it involves general anesthesia and carries a risk of surgical complications, and patients may refuse this procedure due to its invasiveness. Over 73% of the patients in the trial were eligible for diagnostic laparoscopy evaluation at 18 months, yet only 63% of the patients underwent the procedure, indicating that up to 10% of the patients could have had occult PM not captured by the study. Other options to evaluate for PM include imaging, tumor markers, ctDNA, or increasing the follow-up time with conventional imaging, although there is no standardized modality to measure this endpoint.

## 4. HIPECT4 (Spain, 2023)

### 4.1. Trial Design and Results

**Figure 3:**
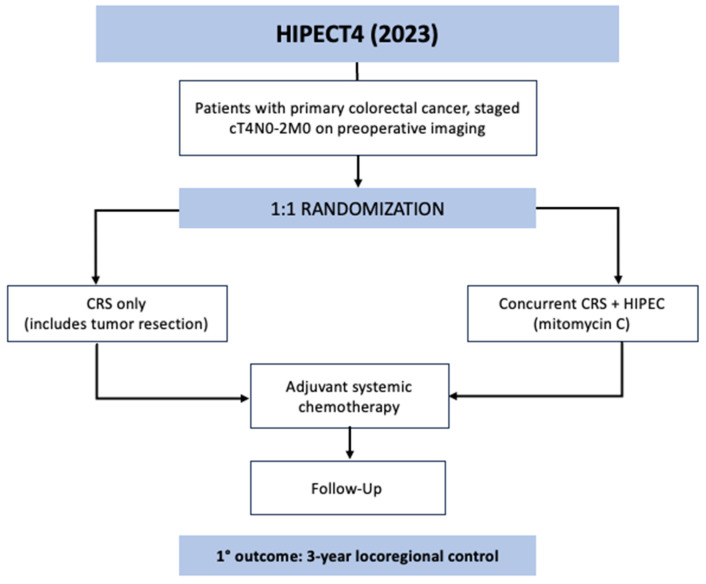
HIPECT4 trial schematic.

HIPECT4 was the most recent phase 3 RCT to evaluate HIPEC for locally advanced colorectal cancer [41]. The trial was conducted across 17 centers in Spain between November 2015 and March 2021. The goal was to assess the efficacy of HIPEC with mitomycin C at the time of resection for T4 colon cancer in controlling locoregional disease. The eligible patients had histologically confirmed colorectal adenocarcinoma that was preoperatively staged at cT4N0-2M0; patients who presented with obstruction or perforation requiring urgent surgery and those with metastases were excluded. All 184 patients were preoperatively randomized 1:1 to the study arm, who underwent CRS and HIPEC with mitomycin C (30 mg/m^2^) for 60 min followed by systemic chemotherapy, or the control arm, who received only CRS followed by systemic chemotherapy (Figure 3). Surgery for all trial participants involved extensive cytoreduction, including complete tumor resection, extensive lymphadenectomy, omentectomy, appendectomy, and possible oophorectomy. The patients in both arms then underwent chemotherapy and were followed every 6 months with imaging and tumor markers. The primary outcome was 3-year locoregional disease control as measured with surveillance imaging. The intention-to-treat analysis showed a 3-year locoregional control rate of 97.6% in the CRS–HIPEC group compared to 87.6% in the CRS-only group (HR 0.21, CI 0.05–0.95, *p* = 0.03). There was no difference in secondary outcomes between groups: 3-year disease-free survival or 3-year overall survival. On analysis of only patients with pathologically confirmed T4 tumors, the CRS–HIPEC group again demonstrated an improved locoregional control rate of 98.3% vs. 82.1% at 3 years (HR 0.09, CI 0.01–0.70, *p* = 0.003). There was no observed increase in morbidity or mortality with the addition of HIPEC in this study.

### 4.2. Strengths and Contributions

This recent study by the Spanish group is the first trial to demonstrate the clinical benefit of prophylactic HIPEC in the setting of locally advanced T4 colon cancer, which carries approximately a 20% risk of developing PM [11,27]. Compared to the previous two trials, the patients in HIPECT4 were the most homogenously selected to include only those with clinical T4 tumors, thereby improving the generalizability of the results for this patient population. The primary endpoint of locoregional recurrence, including the tumor bed and peritoneal surfaces, was selected, appropriately evaluating the efficacy of HIPEC as a regional therapy. Importantly, the authors performed a sub-analysis of the primary outcome using only patients with T4 tumors confirmed on pathology, which strengthens the finding that HIPEC improves disease control within the peritoneum in locally advanced colorectal cancer. Instead of oxaliplatin, this trial used mitomycin C as the HIPEC agent, which works synergistically with heat and has demonstrated good efficacy in CRS–HIPEC for colorectal cancer [60,61]. The perfusion time of 60 min (compared to 30 min with oxaliplatin) also allows for increased drug concentration in tissues and prolonged cytotoxic activity against the residual tumor cells in the peritoneal cavity [19]. Another strength of HIPECT4 is the simultaneous administration of HIPEC at the time of CRS. This strategy is safe without increased complications or toxicity, and it avoids the morbidity and cost associated with a second operation and hospital stay observed in second-look surgery or staged HIPEC.

### 4.3. Limitations and Discussion

One limitation of HIPECT4 is in its preoperative patient selection. While the intended study population was patients with T4 tumors, the trial showed that only 67.9% of preoperatively determined cT4 tumors based on imaging were confirmed to be pT4. This finding is consistent with the literature reporting that high rates of cT4 colorectal tumors are downstaged on pathology [62,63,64] and indicates that 32% of the patients in the trial had <pT4 tumors that may have been overtreated. Unfortunately, given the lack of other reliable diagnostic modalities, preoperative cross-sectional imaging remains the modality of choice for evaluating locally advanced colorectal cancer.

In addition, the study excluded patients who presented with perforated colorectal tumors that required urgent management as the trial design required preoperative randomization, which would not have been feasible in the context of emergency surgery. Moreover, HIPEC is contraindicated in the setting of acute obstruction and perforation [65]. Thus, the 17% identified as perforated tumors in HIPECT4 represents only those with microscopic perforation found on pathologic evaluation [41]. These patient inclusion criteria limit the generalizability of the HIPECT4 results to those with macroscopic perforation of T4 colorectal tumors, who are also at high risk for developing PM [11,27]. Additional studies on early re-exploration with HIPEC for macroscopic perforated colon cancers may be warranted.

A unique feature of this study is the extent of cytoreduction performed for the patients in both trial arms. Although CRS that encompasses complete tumor resection, lymph node dissection, omentectomy, appendectomy, and bilateral oophorectomy in postmenopausal women is prudent in the setting of high-risk T4 tumors, this is not routinely performed. It is, therefore, difficult to compare the locoregional recurrence rates in HIPECT4 with that of other studies, which perform a partial colectomy with local nodal harvest only [64,66,67]. The extensive cytoreduction itself is likely a contributing factor to the study’s overall low recurrence rates [6]; it may also explain why, even after stratifying for only pT4 cancers, the locoregional control in both groups was higher than predicted at the time of trial design (98.3% vs. 82% for the experimental group; 82.1% vs. 64% for the control group).

This study highlights the importance of weighing the benefit of decreased locoregional recurrence with the possibility of overtreating patients with pT2–T3 tumors and risk of complications. A significant advantage of simultaneous CRS with HIPEC is that it requires only one major operation and hospitalization, which not only lowers the cost and total hospital days but also avoids the substantial adhesion burden and complications associated with repeat laparotomy [18,30]. The tradeoff, however, is that patients must be identified preoperatively, and this results in overcapturing and overtreating patients with cT4 disease who will ultimately be downstaged on pathology. While safety and morbidity should be heavily factored into any risk–benefit analysis, this is particularly true when considering prophylactic or risk-reducing treatments. In the HIPECT4 trial, the benefit of CRS–HIPEC in decreasing locoregional recurrence in patients with pT4 colorectal tumors may outweigh the potential overtreatment of those with pT2–T3 disease given the low rate of adverse events associated with the addition of HIPEC.

Lastly, this trial also used surveillance imaging to evaluate local and peritoneal disease recurrence, which lacks sensitivity to detect small PM burden as previously discussed [3,45]. Although the follow-up period of 3 years is relatively long and may allow for PM to grow to a size detectable on CT, some peritoneal disease will remain occult and undetected.

## 5. Implications and Ongoing Trials

Despite their limitations, the negative results from the PROPHYLOCHIP and COLOPEC trials have been discouraging. However, the results from the HIPECT4 trial that reported improved locoregional control with HIPEC have rekindled interest in the potential of intraperitoneal therapies. The three trials discussed in this review are important because they shift the paradigm of using HIPEC as an adjunct to treat PM into using HIPEC as a modality to prevent the development of PM. The impact of HIPECT4 is that it shows the addition of HIPEC with mitomycin C to CRS was effective in preventing local and peritoneal recurrence at 3 years with excellent locoregional control rates. Additionally, the study showed that simultaneous HIPEC at the time of CRS can be safely performed with no increase in adverse events, a bias that many treating oncologists have against the surgical management of peritoneal surface malignancies. Ultimately, demonstrating the efficacy of HIPEC in preventing the development of PM will offer a method to decrease the burden of disease and improve the quality of life in patients with advanced colorectal cancer.

While the renewed interest and research into concurrent and adjuvant HIPEC for colorectal cancer are exciting, one caution to readers is in how to interpret these trials’ results. There is no standard definition for “high-risk colorectal cancer”, and thus, patient inclusion criteria may vary by study. For example, PROPHYLOCHIP included patients with limited resected PM, ovarian metastases, and perforated tumors, which carry a higher risk for developing peritoneal recurrence compared to the HIPECT4 cohort who had T4 tumors without macroscopic perforation or evidence of metastases. A standardized definition of “high-risk colorectal cancer” would be helpful in comparing data across studies. Because of their differing risk profiles for developing PM, patients with T4 tumors, perforated tumors, and resected peritoneal/ovarian metastases should be stratified or evaluated separately in future studies to determine the efficacy of HIPEC in each patient cohort. It is also important to note that the efficacy of mitomycin C versus oxaliplatin as the HIPEC drug in colorectal cancer is unclear. The only trial to compare the two perfusion agents was led by Levine et al. for appendiceal cancer, noting similar survival outcomes between cohorts with a higher rate of leukopenia in the mitomycin C group [68]. However, this study utilized a longer perfusion time at a lower dose for oxaliplatin (120 min, 200 mg/m^2^) when compared to the protocols cited in PROPHYLOCHIP and COLOPEC (30 min, 460 mg/m^2^), and thus, the results may not be directly comparable [18,30,68]. Furthermore, the RCTs lack standardization of outcome measures, such as primary endpoints (PM-free survival vs. overall survival) and mode and timing of diagnosing PM (imaging vs. diagnostic laparoscopy, 1 to 3 years). Therefore, caution should be taken when extrapolating and comparing the results across studies.

A list of current clinical trials that are investigating prophylactic and adjuvant HIPEC in the setting of advanced colorectal cancer is outlined in Table 2.

## 6. Conclusions

In recent years, HIPEC has been studied as a modality to prevent the development of PM in patients with advanced colorectal cancer, although two RCTs had been unable to show a clinical benefit with HIPEC. However, encouraging results from the HIPECT4 trial that showed improved locoregional control with HIPEC have renewed optimism into the therapeutic potential of HIPEC, which will have important treatment implications for these high-risk patients. Ongoing clinical trials will further elucidate the optimal HIPEC regimens and patient populations who will benefit from these interventions.

## Figures and Tables

**Table 1:** Key Characteristics of RCTs on Prophylactic and Adjuvant HIPEC for High-Risk Colorectal Cancer.

Trial	Study Type	Patient Selection	Trial Arms	HIPEC Regimen	Primary End Point and Findings
**PROPHYLOCHIP** (France)	Phase III RCT: 2010–2015 Adjuvant HIPEC	High risk (resected limited PM or ovarian metastases, perforated tumor); 6 months adjuvant chemotherapy with no evidence of recurrence	Surveillance only vs. Second-look surgery with CRS–HIPEC	Oxaliplatin (460 mg/m^2^ or 300–360 mg/m^2^ + irinotecan) for 30 min	3-year disease-free survival No significant difference (53% vs. 44%, *p* = 0.82)
**COLOPEC** (The Netherlands)	Phase III RCT: 2015–2017 Adjuvant HIPEC	Locally advanced or perforated colorectal cancer (clinical or pathologic T4N02M0)	Adjuvant chemotherapy only vs. Adjuvant HIPEC followed by chemotherapy	Oxaliplatin (460 mg/m^2^) for 30 min	18-month PM-free survival No significant difference (76.2% vs. 80.9%, *p* = 0.28)
**HIPECT4** (Spain)	Phase III RCT: 2015–2021 Prophylactic HIPEC	Locally advanced colon cancer (clinical T4N02M0)	CRS only vs. Concurrent CRS–HIPEC	Mitomycin C (30 mg/m^2^) for 60 min	3-year locoregional control (LC) Improved LC rate after HIPEC (97.6% vs. 87.6%, *p* = 0.03)

**Table 2:** Summary of Ongoing Randomized Controlled Trials in Prophylactic and Adjuvant HIPEC for High-Risk Colorectal Cancer.

Trial	Study Type	Projected Inclusion and Enrollment	Trial Arms	HIPEC Regimen	Primary End Point
(China) NCT02179489	Phase III RCT, Adjuvant HIPEC	Colorectal cancer: cT4, perforation, minimal PM, or ovarian metastases; after surgical resection + 6 months chemotherapy 271 total, randomized into 2 arms	Surveillance only vs. Second look surgery with HIPEC	Mitomycin C (30 mg/m^2^) for 60 min	Disease-free survival (3 years)
**APEC Study** (China) NCT02965248	Phase III RCT, Prophylactic HIPEC	Colorectal cancer: any cT4 or cT3 with mucinous or signet ring cell 147 total, randomized	Surgery + adjuvant chemotherapy vs. Surgery with HIPEC + adjuvant chemotherapy	Raltitrexed (3 mg/m^2^) for 60 min or Oxaliplatin (130 mg/m^2^) for 30 min	Rate of PM (3 years)
**CHECK Study** (Italy) NCT03914820	Phase III RCT, Prophylactic HIPEC	Colorectal cancer: cT4, perforation, limited peritumor PM, or ovarian metastases 330 total, randomized into 2 arms	Standard surgery + adjuvant chemotherapy vs. Prophylactic surgery + HIPEC CO_2_	Mitomycin C (35 mg/m^2^) for 60 min	Local recurrence-free survival (3 years)
(China) NCT04370925	Phase III RCT, Prophylactic HIPEC	Colorectal cancer: cT4N0-2M0 without perforation 688 total, randomized into 2 arms	Surgery + adjuvant chemotherapy vs. Surgery with HIPEC + adjuvant chemotherapy	Mitomycin C (30 mg/m^2^) for 90 min	PM-free survival (3 years)
**WUHIPEC02** (China) NCT04845490	Phase II RCT, Prophylactic HIPEC	Colorectal cancer: cT3 or cT4 without metastases 201 total, randomized into 3 arms	Surgery with hyperthermic saline (repeat at 48 h) + chemotherapy vs. Surgery with HIPEC (repeat at 48 h) + chemotherapy	Mitomycin C (30 mg/m^2^) for 60 min or Lobaplatin (50 mg/m^2^) 60 min	PM-free survival (3 years)
**PROMENADE** (Italy) NCT02974556	Phase III RCT, Prophylactic HIPEC	Colorectal cancer: cT3 or cT4 without metastases undergoing curative resection 140 total, randomized into 2 arms	Standard surgery + adjuvant chemotherapy vs. Extensive cytoreduction with HIPEC + adjuvant chemotherapy	Oxaliplatin (460 mg/m^2^) for 30 min	Rate of PM (3 years)
